# Damage of Stone Baskets by Endourologic Lithotripters: A Laboratory Study of 5 Lithotripters and 4 Basket Types

**DOI:** 10.1155/2013/632790

**Published:** 2013-10-30

**Authors:** Jens Cordes, Felix Nguyen, Birgit Lange, Ralf Brinkmann, Dieter Jocham

**Affiliations:** ^1^Clinic of Urology, Luebeck Campus, University Medical Center Schleswig-Holstein, Ratzeburger Allee 160, 23538 Luebeck, Germany; ^2^Medical Laser Center Luebeck GmbH, Ratzeburger Allee 160, 23538 Luebeck, Germany

## Abstract

*Background*. In some cases, the ureteral stone is simultaneously stabilized by a stone basket when endourologic lithotripsy is performed. This stabilization can be either on purpose or by accident. By accident means that an impaction in the ureter occurs by an extraction of a stone with a basket. A stabilization on purpose means to avoid a retropulsion of the stone into the kidney during lithotripsy. At this part of the operation, stone baskets have been frequently damaged. This severing of wires can lead to ureteral trauma because of hook formation. *Material and Methods*. In a laboratory setting, the time and the pulse numbers were measured until breaking the wires from four different nitinol stone baskets by using five different lithotripsy devices. The endpoint was gross visibledamage to the wire and loss of electric conduction. *Results*. The Ho:YAG laser and the ultrasonic device were able to destroy almost all the wires. The ballistic devices and the electrohydraulic device were able to destroy thin wires. *Conclusion*. The operating surgeon should know the risk of damagefor every lithotripter. The Ho:YAG-laser and the ultrasonic device should be classified as dangerous for the basket wire with all adverse effects to the patient.

## 1. Introduction

In the past years, the number of endoscopic procedures in western countries has increased [1, 2]. One endoscopic procedure is endourological lithotripsy, in which the stone is destroyed within the ureter. Sometimes the stone is simultaneously stabilized by a stone basket.

This stabilization could be on purpose or by accident. By accident means that an impaction in the ureter occurs by an extraction of a stone with a basket. A stabilization on purpose means to avoid a retropulsion into the kidney during a lithotripsy.

At this part of the operation, stone baskets have been frequently destroyed [3]. This severing of wires can lead to ureteral trauma because of hook formation [4]. On the other hand, it could release the impacted stone from the basket by serving all wires of the basket [5]. 

Fragmentation of stone baskets is well known as a result of using the Ho:YAG laser. For other lithotripters, there exists just one study until now [6].

Baskets with a diameter of 3 F were destroyed by lasers in 15 to 34 seconds, and tipless baskets (1.8 F diameter) were destroyed in 1 to 4 seconds with pulse energy of 0, 8, and 2 J at a pulse frequency of 5 Hz. The guidance of the optical fiber occurred by means of a cystoscope in a basin filled with water (Honeck et al., 2006) [7]. Cordes et al. [6] confirm these results in an artificial model through a renoscope. They also showed that a semirigid ultrasonic lithotripter could destroy the baskets in the set time limit of one minute. Only plaited baskets could resist ultrasonic force. In that study, ballistic lithotripters as the Swiss Lithoclast EMS or the Lithorapid EL-28 Olympus could not destroy one basket. An electrohydraulic device was not tested. For the fragmentation of stones, there are various lithotripters available which can be used through a semirigid renoscope. Electrohydraulic and ultrasonic lithotripters were first used before 1980 to fragment bladder stones [8, 9] and since the early 1980s have been modified for use via semirigid renoscopes [10]. The ultrasonic device has limitations which include its inability to fragment hard stones composed of calcium oxalate and the difficulty in miniaturizing the probe [11, 12]. Problems of the powerful electrohydraulic device are its narrow safety margins and a high incidence of ureteral injury [13, 14]. In the 90s, two ballistic lithotripters were introduced. The lithoclast was presented by Lanquetin et al. [15] and Denstedt et al., [16] and the electrokinetic lithotripter was introduced by Vorreuther et al. [17] and Schulze et al. [18]. In a randomized *in situ* trial by Menezes et al., there was no significant difference between these ballistic lithotripters with regard to the stone-free rate, procedure duration, fragmentation time, proximal stone migration rate and equipment failure [19].

The first preliminary experience with the holmium:YAG laser lithotripsy was performed in the mid-90s [20]. The primary mechanism of lithotripsy is photothermal [21]. There are no significant photoacoustic effects [21]. Holmium:YAG yields smaller fragments compared to electro-hyraulic lithotripsy and mechanical lithotripsy, so that fragments are more likely to pass without a problem [22]. Ho:YAG laser therapy is named as the gold standard lithotripsy modality for endoscopic lithotripsy [23].

In the 70s, Enrico Dormia invented and tested his stone basket. It was first used under cystoscopic and radiologic control [24]. In 1982, Huffman et al. presented an initial series of stone extraction under the vision of an ureteroscope using a 5 F stone basket [25]. Limitations of the use of stone basket extraction postulated from Dormia 2000 are as follows: a stone bigger than 1 cm, a stiff ureter (as in Ormond or postradiotherapy stiffness), and the presence of stenosis [26].

Today, in our clinic, we try to be as atraumatic as possible. If there is any doubt in being atraumatic, we try to do a lithotripsy after releasing the stone from the basket or when it is not possible in the basket. 

Our study now shows if and how quick ballistic, electro-hydraulic, ultrasonic, and laser lithotripters destroy basket wires in a strict laboratory setting. As far as we know, it is the first comparing study of these five lithotripters and four baskets.

## 2. Material and Methods

The lithotripters were a Ho:YAG laser (Vera Pulse, Coherent), an electrokinetic-ballistic device (Lithorapid EL-28, Olympus), a pneumatic-ballistic device (Swiss Lithoclast, EMS), an ultrasonic lithotripter (Calcuson 27610029, Storz), and an electrohydraulic device (Riwolith 2137, Wolf).

The adjustment of the lithotripter corresponded to the adjustments customary for endoscopic lithotripsy and recommended by the manufacturer. For the holmium:YAG laser, this meant a pulse energy of 0.8 J at a pulse frequency of 8 Hz. The diameter of the optical fiber was 365 *μ*m. The pneumatic-ballistic lithotripter had an adjustment of 1.5 bar, which corresponds to an output of approximately 0.63 J with 50 Hz. The probe had a diameter of 1 mm. The electrohydraulic device was used with 4-5 pulses per second for 3 seconds at level one (levels 1–3). The probe had a diameter of 5 F. The ultrasonic device was operated at the middle level 2 (levels 1–3) with a probe thickness of 1.5 mm. Level B, which corresponds to the middle level with approximately 0.55 J and 15 Hz, was selected with the electrokinetic device. The electrode had a diameter of 3 F.

**Figure 1:**
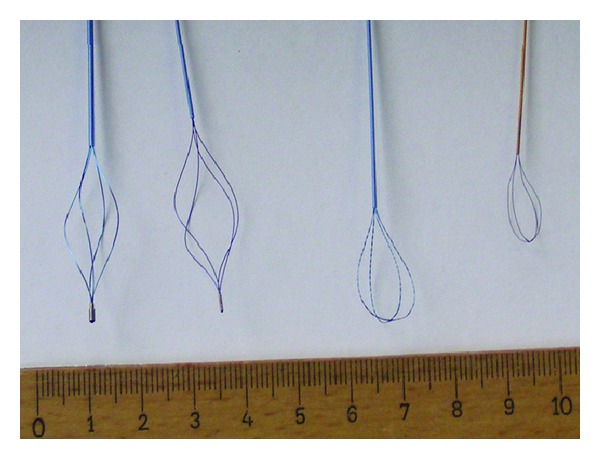
All four different basket types from left to right are as follows: (i) Dormia (Mentor Porgès), 4 helical wires (diameter 0.25 mm), 3.5 F, nitinol, (ii) Dormia (Mentor Porgès), 4 helical wires (diameter 0.18 mm), 2.5 F, nitinol, (iii) Epflex, tipless, plaited basket (wire diameter 0.127 mm), 2.5 F, nitinol, and (iv) Equadus (OptiMed), tipless basket, 4 wires (diameter 0.07 mm), 1.8 F, nitinol.

The stone baskets examined included (Figure 1).

- Dormia (Mentor Porgès), 4 helical wires (diameter 0.18 mm), 2.5 F, nitinol;
- Dormia (Mentor Porgès), 4 helical wires (diameter 0.25 mm), 3.5 F, nitinol;
- Equadus (OptiMed), tipless basket, 4 wires (diameter 0.07 mm), 1.8 F, nitinol;
- Epflex, tipless, plaited basket (diameter 0,127 mm), 2.5 F, nitinol.

**Figure 2:**
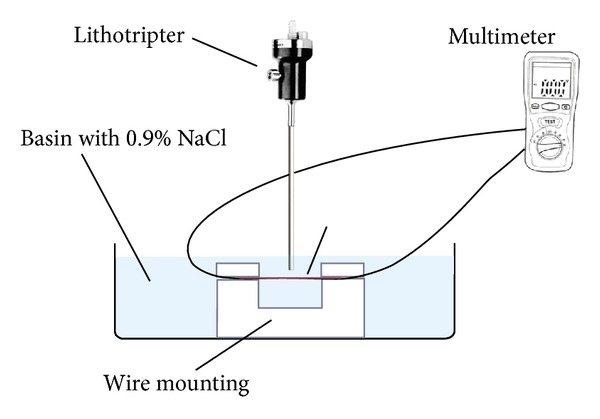
The schematic drawing of the experimental setup. The basket wire is fixed in a holding instrument under water. The incident angle of the lithotripter is 90°. The ohmic resistance of the basket wire is measured by an ohmmeter.

The baskets were used once before this experiment during an extraction of a stone and subjected to a visual inspection after cleaning. The experimental setup (Figure 2) was a strict laboratory setting. The wires were locked in a special holding instrument under water, and the probes incident angle was 90°.

The time and the pulses until destruction were measured and documented by video. The endpoint was gross visible damage to the wire and loss of electric conduction. It was measured by a Multimeter (2010 DMM, Peaktech, Ahrensburg, Germany). 

The modus, called “Durchgangsprüfung mit Summer” in which a permanent sound signal indicates an intact conduction and a loss of this signal shows an interruption of conduction.

## 3. Result

**Figure 3:**
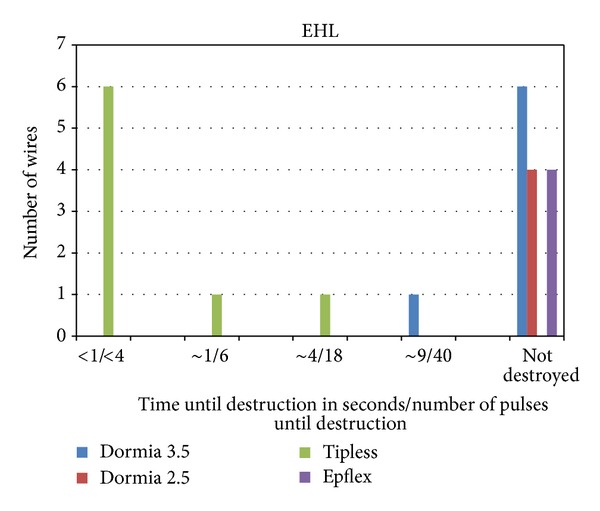
The EHL destroyed all tipless baskets wires in a range from 1 to 4 sec/fewer than 4 to 18 pulses. The one 3,5 Dormia basket was destroyed after 9 sec/40 pulses.

The electrohydraulic device destroyed all eight tipless basket wires 1.8 Ch from 1 to 4 sec/fewer than 4 to 18 pulses and one tipped basket 3.5 Ch after 9 sec/40 pulses (Figure 3).

**Figure 4:**
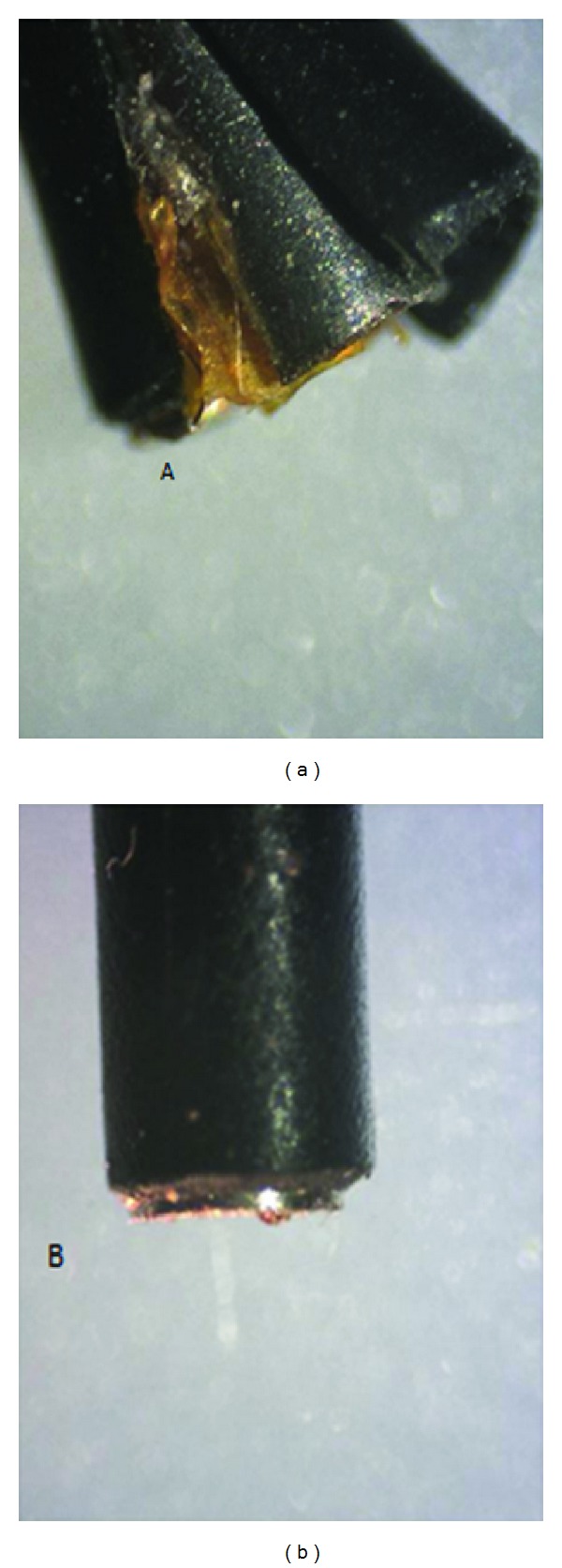
Destroyed (a) and intact (b) probes of the electrohydraulic device (magnification of 60).

Beside destroying these six from 23 baskets wires four probes of the electrohydraulic device were destroyed (Figure 4). This shows that the electrohydraulic device has a minimal potential for destroying basket wires (Table 1).

**Figure 5:**
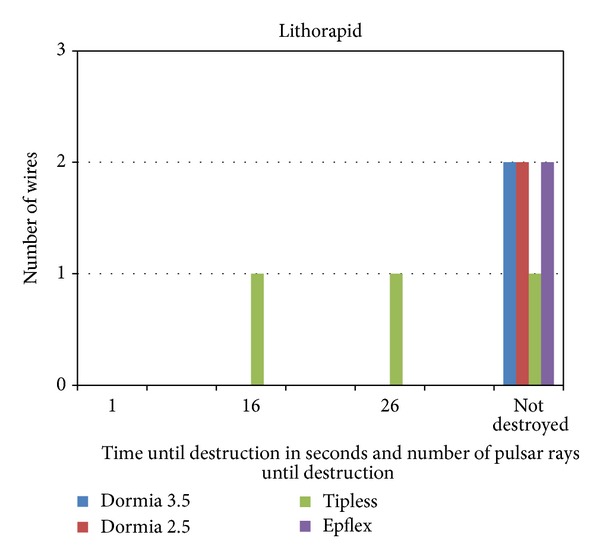
Electrokinetic device (Lithorapid) with destruction of two 1.8 F tipless baskets after 16 or 26 pulses and seconds.

The electrokinetic device destroyed two 1.8 F tipless baskets after 16 and 26 sec or pulses. The other basket wires stayed unfragmented (Figure 5), so that the electrokinetic device is in the same category as the electrohydraulic device (Table 1).

**Figure 6:**
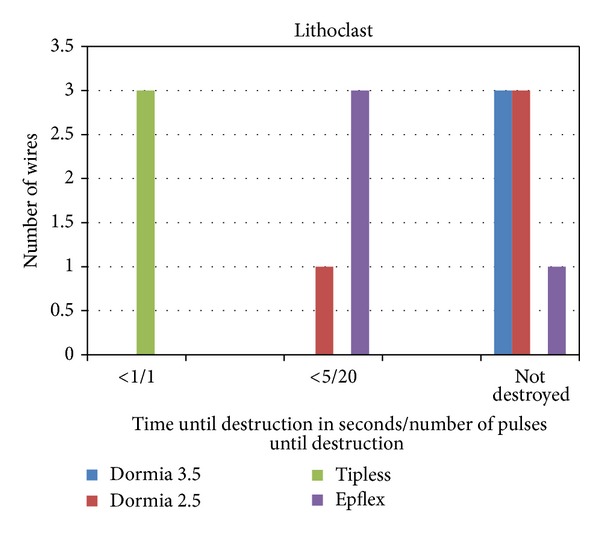
Pneumatic-ballistic device (Lithoclast) for the destruction of all 1.8 F tipless baskets, 3 of four 2.5 F tipless baskets, and one of four 2.5 F Dormia baskets. For the 1.8 F tipless baskets, the device just needed less than one second/one pulse. For the bigger wires, the device needed fewer than 5 sec/20 pulses.

The pneumatic-ballistic device destroyed all the three 1.8 F tipless, 3 of four 2.5 F tipless, and one of four 2.5 F Dormia basket wires. The 3.5 F Dormia basket was not destroyed. For the 1.8 F tipless basket the device just needed one pulse for destruction. For the bigger wires, the device needed 20 pulses fewer than 5 sec (Figure 6). The resume for this device is that it is a little bit more potential as the devices before described but it is still categorized in the group of minimal danger for the wire (Table 1). 

**Figure 7:**
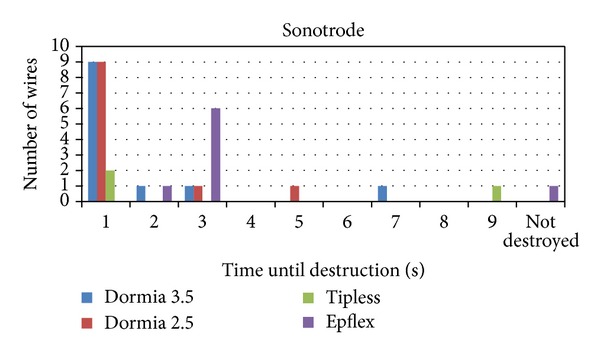
The ultrasonic lithotripter destroyed all baskets wires with the exception of one 2.5 F tipless basket. The destruction took place from 1 to 9 sec.

The ultrasonic lithotripter destroyed all baskets wires with the exception of one plaited, 2.5 F, tipless basket. The destruction took place from 1 to 9 sec (Figure 7). This device is clearly one with danger for the basket wire (Table 1).

**Figure 8:**
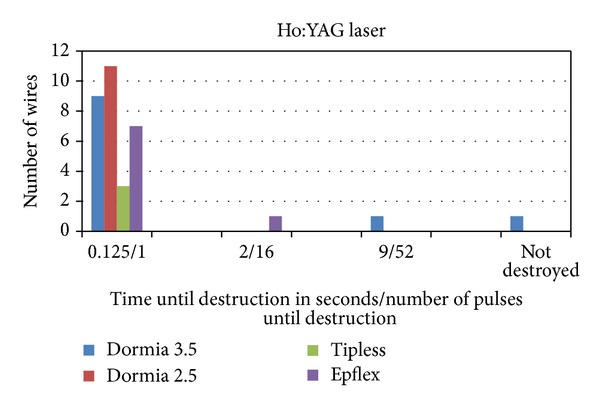
The Hol:YAG laser destroyed all baskets wires with the exception of one 3.5 F Dormia basket. The destruction took place from 1 to 9 sec/from 1 to 52 pulses mostly after one pulse.

The Hol:YAG laser destroyed all baskets wires with the exception of one 3.5 F Dormia basket. It took place from 1 to 9 sec or one to 52 pulses mostly after 1 pulse (Figure 8). As expected, the laser falls into the same category as the ultrasonic device (Table 1).

**Figure 9:**
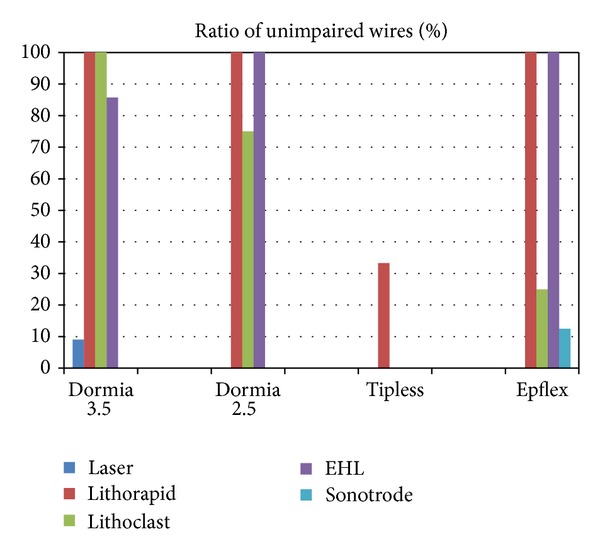
Over all the four basket types, the tipless 1.8 CH basket has the lowest resistance.

**Figure 10:**
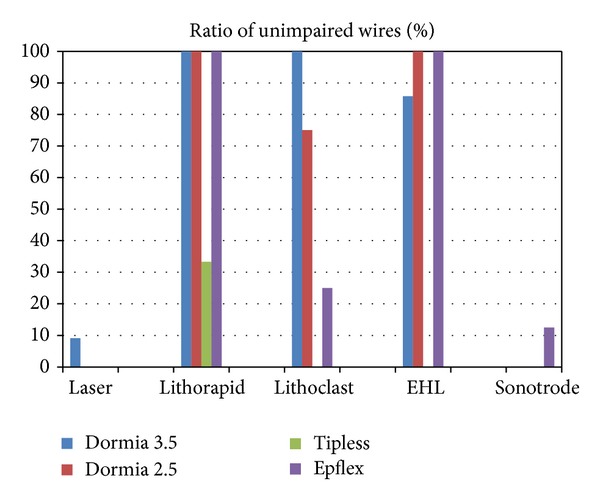
Over all the lithotripters, the laser and the ultrasonic device have the lowest ratio of unimpaired wires.

Also as expected the weakest basket was the 1.8 Ch tipless basket (Figure 9). What is interesting and new is that, beside the laser, the ultrasonic device destroyed most basket wires (Figure 10) and the electrohydraulic device is a minimal danger for the basket wire.

## 4. Discussion

For the lithotripsy in the basket, one should know the interaction of the lithotripter and the basket. This interaction was already explored by Cordes et al. [6] in a model related to the clinical situation. In this study, the sonotrode and the laser could destroy basket wires, and the ballistic instruments could not destroy basket wires in the set time limit of one minute. This new finding needed further investigation.

The ballistic devices as the pneumatic and the electrokinetic lithotripters have the potency of destroying the wire in this study. However, this potency is not clinically relevant as Cordes et al. showed [6]. This is due to the inflexible setting of this study, where the wire is fixed in a holding instrument. We would classify these two lithotripters as minimal dangerous for the basket wire, because in the clinical setting the basket with the stone is flexible, and Cordes et al. [6] showed that just the stone was destroyed (Table 1).

What is new is that in this inflexible setting the electrohydraulic device just destroyed the basket wires of all tipless baskets 1.8 Ch and just one wire of the 3.5 Ch basket, so that one can classify it as minimal dangerous for the wire (Table 1).

In a study by Piergiovanni et al. [14], in which four lithotripters were comparatively examined (EMS Swiss LithoClast, Olympus LUS ultrasonic device, Walz Lithotron EL 23, and Coherent Vera Pulse holmium:YAG laser), a different potency of bladder and ureteral lesions was shown to apply in the pig model. The ultrasonic device and the Lithoclast were categorized in the group of minimal dangerous lithotripters. The holmium:YAG laser and the electro-hydraulically functioning device were categorized in the group of potentially dangerous lithotripters, which can bring about a perforation (Table 1).

In our strict laboratory study, the potency of destroying the basket by the laser and by the ultrasonic device could be confirmed. We would classify them as maximal dangerous for the wire; see Table 1. Also, what is new is that the electrohydraulic device has minimal danger for the basket wire.

The limitation of this study is using an *in vitro* model with a fixed wire using an incidence angle of 90°. This is also shown in the experimental study of Freiha et al., in which a guidewire damage by laser varied with the inverse of the cosine of the incident angle [27].

## 5. Conclusion

For further investigations, we recommend that the model for testing lithotripters and basket should be a “clinical model” as that which Cordes et al. [6] applied for, in which the baskets were tested through a renoscope under water in a catheter.

We think that knowing which potency of danger distinct lithotripters could produce is very important for the operating surgeon. This should be related to the clinical advantage, which can be provided by different lithotripters. For example, it is well known that the Ho:YAG Laser has a higher stone-free rate, a diminished operation time, and a diminished rate of double J stent insertion [28].

## Figures and Tables

**Table 1:** Potential Dangers of Lithotriptors: Danger for the ureter tested by Piergovanni et al. [14] (not testing the Electrokinetic device). Danger for the basket wire tested by Cordes et al. [6] (not testing the electrohydraulic device).

	Danger for Basket wire	Minimal danger for Basket wire
Danger for Ureter	Ho : YAG [6, 14]	Electrohydraulic device [14]

Minimal danger for ureter	Ultrasonic device [6, 14]	Pneumatic ballistic device [6, 14], (Electrokinetic device [6])
